# Study on metabolites of *Bacillus* producing soy sauce‐like aroma in Jiang‐flavor Chinese spirits

**DOI:** 10.1002/fsn3.1266

**Published:** 2019-11-25

**Authors:** Xingxiu Zhao, Yuanhao Liu, Li Shu, Yiguo He

**Affiliations:** ^1^ College of Bioengineering Sichuan University of Science and Engineering Zigong Sichuan China

**Keywords:** Chinese spirits, GC‐MS, headspace solid‐phase microextraction (HS‐SPME), pyrazines, solid‐phase microextraction, soy sauce flavor

## Abstract

Jiang‐flavor Chinese spirits are one of the four basic liquor types in China. It has thousands of years of history in China and is widely enjoyed because of its unique flavor and broad application. Although Jiang‐flavor Chinese spirits have a unique soy sauce flavor, the associated key compounds and production mechanism remain unknown. To investigate this process, soy sauce flavor‐producing strains were obtained, and their metabolites were evaluated. Using wheat as the fermentation medium, we observed changes in total acid, amino nitrogen, and reducing sugar in three strains of *Bacillus cerberus* with high yield of tetramethylpyrazine during the fermentation process. The results showed that total acid and amino nitrogen contents increased and reducing sugars decreased in a time‐dependent manner. Additionally, detection of volatile compounds via solid‐phase microextraction and gas chromatography–mass spectrometry that total pyrazine content reached 43.175%, 50.461%, and 45.955% in wheat fermentation medium fermented by strains Q1, Q2, and Q5, respectively, suggesting that this important flavor compound might be related to the flavor of soy sauce. Moreover, we found that fermentation time was an important factor in soy sauce flavor, as volatile compounds were detected at different times by the three strains, with pyrazines not detected before 48 hr and peaking at 50.461% after 144 hr. These results indicated that strain Q2 exhibited optimal fermenting performance and might be useful for fermentation of Jiang‐flavor Chinese spirits.

## Graphical abstract

Although Jiang‐flavor Chinese spirits have a unique soy sauce flavor, the associated key compounds and production mechanism remain unknown. To investigate this process, soy sauce flavour‐production strains were obtained, and their metabolites were evaluated.

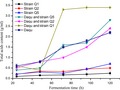

## 1 INTRODUCTION

Jiang‐flavor Chinese spirits are one of the four basic liquor types in China. It has thousands of years of history in China and is widely enjoyed because of its unique flavor and broad application. The unique soy sauce flavor and roasted aroma are the result of complex biochemical processes by microorganisms involved in the fermentation process; however, the key components related to the flavor of Jiang‐flavor Chinese spirits remain unclear due to its complex composition and low content of aroma components (Xiong, 1994; Zhu et al., 2007). Pyrazines are created via Maillard reactions (Oh, Hartman, & Ho, 1992), with recent studies reporting that *Bacillus subtilis* is capable of producing pyrazines during soybean solid‐state fermentation (Besson, Creuly, Gros, & Larroche, 1997; Larroche, Besson, & Gros, 1999). Pyrazines mainly present the flavor of black bean and nut and are the main source of characteristic flavor of fermented soybean products. Proteomics studies have also shown that the presence of methoxypyrazines in these compounds improves the flavor of red wine and the same effect in Chinese spirits (Zhu et al., 2007). This suggests that pyrazines might be among the most important aroma compound in soy sauce flavor liquor (Shen, 1997, 2002, 2003).

During this study, we obtained three *Bacillus* strains (Q1, Q2, and Q5) from high‐temperature Daqu, with each capable of producing strong Jiang‐flavor in wheat fermentation medium and similar to that of Jiang‐flavor Chinese spirits. The volatile flavor components of the Jiang‐flavor fermented by the strains were detected by headspace solid‐phase microextraction (HS‐SPME) and gas chromatography–mass spectrometry (GC‐MS), with the results indicating high pyrazine content. The results suggest that the Maotai flavor originated from the bacteria producing the key soy sauce flavor compound and enables further analysis of the metabolic mechanisms associated with the production of key soy sauce flavor compounds to improve the manufacturing process of Jiang‐flavor Chinese spirits.

## 2 MATERIALS AND METHODS

### 2.1 Media preparation

LB medium was prepared as follows: peptone 10 g, beef extract 3 g, sodium chloride 5 g, agar 15 g, and ddH_2_O 1,000 ml (pH 7.2), followed by autoclaving at 121°C for 20 min. Screening medium was prepared as follows: glucose 50 g, peptone 50 g, and K_2_HPO_4_ 20 g (pH 7.0), followed by autoclaving at 121°C for 20 min. Fermentation medium was prepared as follows: Wheat grains were crushed and soaked in the same volume of water (w/v) for 18 hr, followed by autoclaving at 121°C for 30 min.

### 2.2 Strain isolation and culture conditions

Daqu is a saccharifying and fermenting agent with a significant impact on the flavors of the products. It can be categorized according to maximum incubation temperatures (high, medium, and low) and flavor (sauce, strong, light, and miscellaneous) (Zheng, Tabrizi, Nout, & Han, 2011). The content of tetramethylpyrazine was higher in liquor brewed by Daqu at high temperature (Fan, Xu, & Zhang, 2007). Strains producing soy sauce flavor were isolated from high‐temperature Daqu obtained from Langjiu Liquor production factory (Luzhou, China). The sample of Jiang‐flavor high‐temperature Daqu (25 g) was added to 225 ml of sterilized ddH_2_O and shaken for 30 min, followed by heat treatment of the cell suspension in a water bath at 80°C for 20 min to kill cells and enrich endospores. After serial dilution and coating plates, bacteria exhibiting good growth and different colony morphology were purified by the streak plate method, and each isolated colony was inoculated into LB medium and stored at 4°C. According to the pyrazine metabolic pathway, 3‐hydroxy‐2‐butanone (HB) is the key precursor for pyrazine synthesis; therefore, screened strains were inoculated into LB medium, and the fermentation broth was assayed to determine its HB‐forming ability using the Voges‐Proskauer (V‐P) test. Additionally, screened strains were inoculated into 50 g of fermentation medium and cultured at 35°C for 2 days, followed by additional culture at 45°C for 2 days and 55°C for 2 days. Strains producing soy sauce flavor were screened according to their sensory properties, with strains Q1, Q2, and Q5 identified as *Bacillus cerberus*.

### 2.3 Solid fermentation process to produce soy sauce flavor

Strains and Daqu were inoculated in medium containing 50 g of wheat and cultured at 35°C for 2 days, followed by additional culture at 45°C for 2 days and 55°C for 2 days. The fermentation process is described in Table 1. The total acid, amino nitrogen, and reducing sugars were determined every 24 hr during the fermentation process.

**Table 1:** Fermentation process

Number	Daqu (g)	Strains (mL)		
		Q1	Q2	Q5
1	5	0	0	0
2	5	2	0	0
3	5	0	2	0
4	5	0	0	2
5	0	2	0	0
6	0	0	2	0
7	0	0	0	2

### 2.4 Chemical and quantitative analyses during the fermentation stage

#### 2.4.1 Sample preparation

Samples (10 g) were periodically taken at 24, 48, 72, 96, and 120 hr during fermentation and mixed with 10 ml of distilled water to a constant volume of 100 ml, followed by filtering. The obtained filtrate was used to determine the total acid, amino nitrogen, and reducing sugar contents.

#### 2.4.2 Determination of total acid content

Total acid was determined by titration using a previously described method (Ministerio de Sanidady Consumo, 1985). Briefly, the diluted sample (10 ml) was mixed with 40 ml of distilled water and titrated to pH 8.2 with 0.1 N NaOH. The volume of consumed NaOH was recorded to allow measurement of total acidity according to the following calculation (1):

(1) $$Y=\left(C\;\times \;V\;\times \;K\right)/V_{0}$$

where *Y* is the total acid content of the samples (g/mL), *C* is the concentration of NaOH standard solution (mol/mL), *V* is the volume of NaOH consumed by titration (mL), *V*_0_ is the volume of titrated sample solution (mL), and *K* is the conversion coefficient (0.060). Here, 1 ml NaOH standard solution was equivalent to the quality of acetic acid.

#### 2.4.3 Determination of amino nitrogen content

Amino acid nitrogen content was measured according to a previously described titration method (Cai & Yuan, 1982). Formalin solution (10.0 ml of 40% formalin solution) was added to the sample, mixed, and titrated with 0.1 N NaOH to pH 9.2, with the volume of consumed NaOH recorded. Simultaneously, 100.0 ml of H_2_O was adjusted to pH 8.20 with NaOH, followed by the addition of 10.0 ml of 40% formaldehyde solution and titration to pH 9.20 with 0.1 N NaOH. According to the consumption of NaOH (mL), the content of amino acid nitrogen was calculated as follows (2):

(2) $$Y\%=\left[\left(V_{1}-V_{2}\right)\;\times \;C\;\times \;0.014\;\times \;100\right]/\left(m\;\times \;10/100\right)$$

where *Y* is the amino acid nitrogen content of the samples, *V*_1_ is the volume of NaOH consumed during sample titration (mL), *V*_2_ is the volume of NaOH consumed by blank titration (mL), *C* is the concentration of NaOH standard solution (M), m is the sample weight (g), and 0.014 represents the molar mass of the nitrogen (g/mmol).

#### 2.4.4 Determination of reducing sugar content

Reducing sugars were determined by the Fehling method (Chemists & Chemists, 1920; Fadda & Mulas, 2010). Briefly, standard solution containing 5 ml of Fehling A, 5 ml of Fehling B, and 10 ml distilled water was titrated by adding a previously prepared sugar solution until discolouration. Reducing sugar content was calculated as follows (3):

(3) $$W=250\;\times \;m_{2}/\left(m\;\times \;V\;\times \;1000\right)$$

where *W* is the reducing sugar (glucose) content of the samples (%), *m* is the sample weight (g), *m*_2_ is the standard solution (containing Fehling A and B) equivalent to the content of the reducing sugar (glucose; mg), and *V* is the average volume of consumption of the sample solution (mL).

### 2.5 Analysis of volatile flavor compounds

#### 2.5.1 HS‐SPME

HS‐SPME and GC‐MS were performed using a 50‐/30‐μm divinylbenzene/carboxen fibers purchased from Supelco Inc. (Bellefonte, PA, USA). Before analysis, the fiber was conditioned for 2 hr by inserting it into an Agilent 6890–5975 gas chromatograph (Agilent Technologies, Santa Clara, CA, USA) at 250°C to prevent pollution. Each sample following a 6‐day fermentation (3 g) was, respectively, placed in a 15‐mL phial at room temperature along with a magnetic stirrer and tightly covered with a Teflon‐faced silicone septum. The sample was balanced for 10 min at 60°C in a water bath and extracted at the same temperature with continuous stirring for 50 min. After extraction, the fiber was pulled into the needle sheath, the SPME unit was removed from the phial and inserted into the injection port of the gas chromatograph, and the fiber was desorbed for 5 min at 250°C. All experiments were performed in triplicate.

#### 2.5.2 GC‐MS analysis

Compound identification was performed on an Agilent 6,890 gas chromatography and a 5,975 mass spectrometer (Agilent Technologies) using helium as the carrier gas and a flow rate of 1 ml/min. Separation was performed on a DB‐WAX 30‐m × 0.25‐mm × 0.25‐μm capillary column (Agilent Technologies) at a furnace temperature of 50°C for 2 min, heating to 280° at a heating rate of 5°C/min and holding for 2 min. The electron collision energy was 70 eV, and the ion source temperature was 230°C. Chromatographic records of total ion currents in the mass range of ~ 10 to ~ 550 were monitored.

#### 2.5.3 Data analysis

Data were analyzed by MSD Productivity ChemStation data analysis software (version G1701DA; Agilent Technologies). Identification of volatile compounds was performed by comparing their mass spectra with those of the National Institute for Standards and Technology (search version 2.0) and the Pesticides Retention Time Lock (RTLPEST; part no. G1672AA; version A.03.00) mass spectral library. Determination of percentage composition was based on peak area normalization (the area of a given peak was expressed as a percentage of the sum of the areas of all the peaks) without using correction factors.

## 3 RESULTS AND DISCUSSION

### 3.1 Sensory characteristic of strains producing soy sauce flavor

Q1, Q2, and Q5 were selected as the target strains efficiently producing soy sauce flavor. We simulated the fermentation conditions used during the manufacturing of Maotai‐flavor liquor by using wheat in the fermentation medium. Each strain was cultured for 5 days, and the produced flavor was evaluated. As shown in Table 2, fermentation without strains inoculated in wheat medium exhibited only original material flavor. We subsequently found that different strains Q1, Q2, and Q5 were capable of producing soy sauce flavor, with Q2 producing stronger soy sauce flavor than strains Q1 and Q5. V‐P reaction results showed that all three strains were V‐P‐positive, with Q2 showing the maximum absorbance at 560 nm (A560) and producing a strong flavor.

**Table 2:** Sensory properties of strains producing soy sauce flavor

Sensory properties	Strains			
	None	Q1	Q2	Q5
Flavor	Original material flavor	Soy sauce flavor	Strong soy sauce flavor	Soy sauce flavor
Color	Material color	Light brown	Brown	Dark brown

### 3.2 Changes in total acid content during fermentation

**Figure 1:**
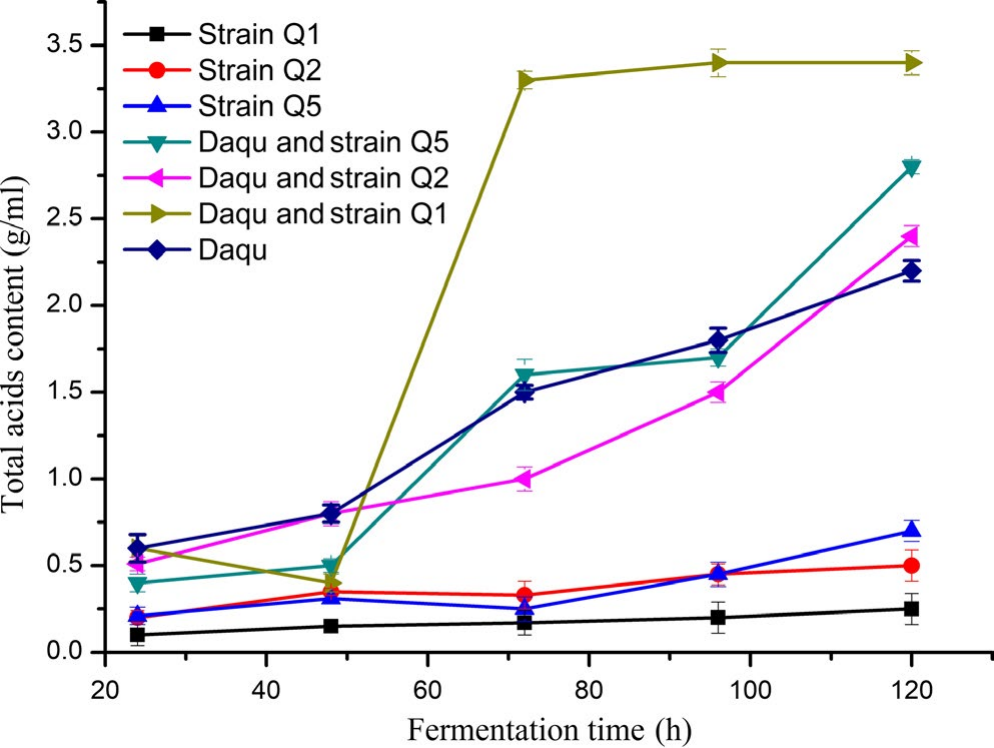
Changes in total acid content during fermentations

According to China National Standard GB/T 26760‐2011, the total acid content in excellent grade high‐alcohol liquor is > 1.4 g/L, and in excellent grade low‐alcohol liquor is > 0.8 g/L. Total acid content in the samples generated in this study is shown in Figure 1. The results showed that total acid content increased along with fermentation time differed significantly between samples and peaked during a 72‐hr fermentation. The total acid contents of the Daqu and strain Q2 were higher than that observed in the other samples, with the total acid produced by strain Q2 and Daqu during fermentation higher than that produced by Daqu fermentation. This result suggested that strain Q2 might produce a large amount of organic acid during fermentation, resulting in the increased total acid content in the fermentation products. Therefore, we used Q2 as the target strain showing high acid production.

### 3.3 Changes in amino nitrogen content during fermentation

**Figure 2:**
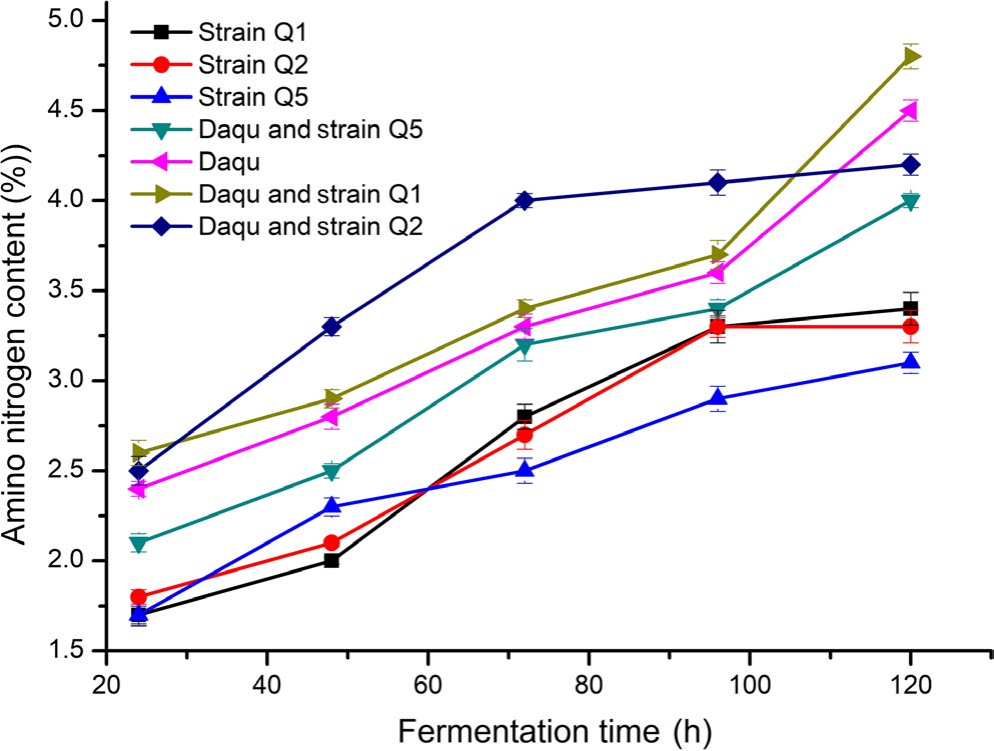
Changes in amino nitrogen content during fermentation

During the fermentation of soy sauce, nitrogen constituents are among the important parameters used to judge the quality of soy sauce. According to the Chinese National Standard (Xu, 2000), Grade A soy sauce should contain total nitrogen and amino nitrogen contents of > 1.30 g/100 ml and > 0.70 g/100 ml, respectively. As shown in Figure 2, we observed a rapid increase in total amino nitrogen content along with fermentation time; however, the contents of total nitrogen and amino nitrogen were both higher in soy sauce fermented by Daqu and strain Q2 than those in the other samples. Additionally, these contents produced by strain Q2 and Daqu during fermentation were higher than those produced by Daqu fermentation. This finding suggested that strain Q2 might produce a large number of proteases capable of hydrolyzing proteins into amino acids, resulting in gradual increases in amino acid nitrogen content.

The content of free amino acids is closely related to the flavor of Jiang‐flavor Chinese spirits, and changes in amino acid nitrogen content reflect gradual increases in free amino acid content. The Maillard reaction occurs between amino compounds and reducing sugars and represents a primary source of food flavor. As amino nitrogen content increases, this also increases soy sauce flavor. These results suggested Q2 as a target strain capable of high amounts of amino nitrogen production.

### 3.4 Changes of reducing sugar contents during fermentation

**Figure 3:**
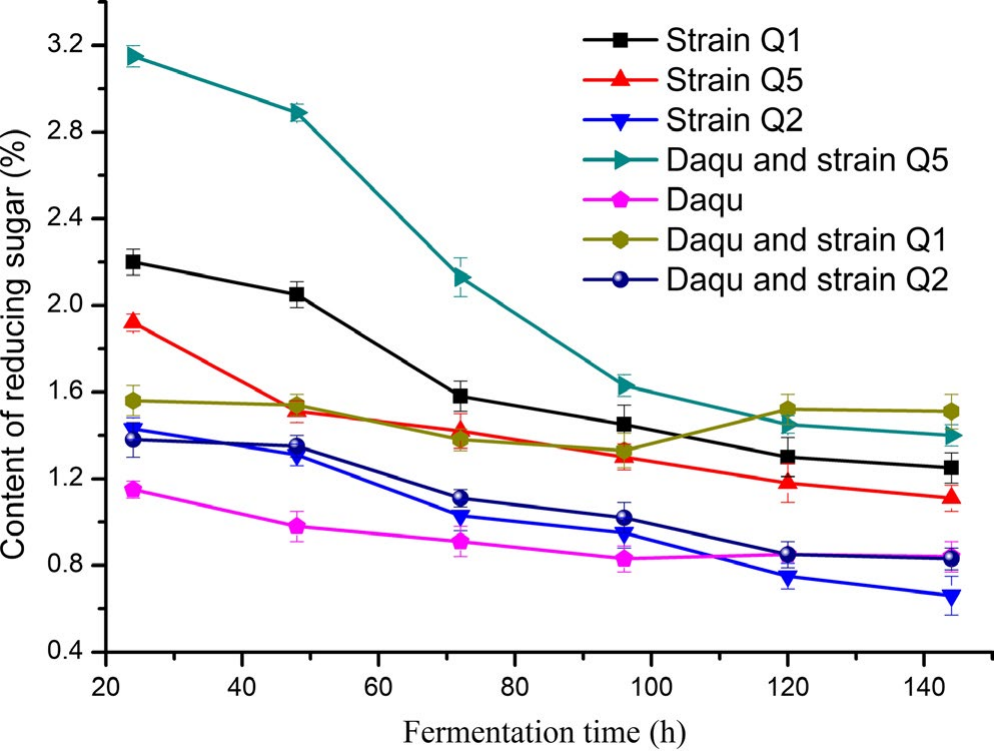
Changes in reducing sugar content during fermentation

The reducing sugar content in the samples is shown in Figure 3, revealing decreases along with fermentation time. This suggested that during the fermentation process, reducing sugars were constantly used by microorganisms.

### 3.5 Volatile flavor profiles in soy sauces fermented for 144 hr

The influence of the target strains on volatile flavor compounds in fermented soy sauce is described in Table 3. A total of 17 volatile compounds, including acids, alcohols, aldehydes, esters, alkanes, ketones, benzene‐ring compounds, and pyrazines, were identified in soy sauces fermented by strains Q1, Q2, and Q5, with a total of 12, nine, and 10 volatile compounds found, respectively. Additionally, we found the volatile compounds HB (No. 8), 2,3‐butanediol (No.10), 2,5‐dimethyl pyrazinel (No. 11), 2,3,5‐trimethylpyrazine (No. 12), and 2,3,5,6‐tetramethylpyrazine (No. 13) in soy sauces fermented by strains Q1, Q2, and Q5, among which pyrazine content reached the highest percentage (43.175%, 50.461%, and 45.955%) in soy sauces fermented by strains Q1, Q2, and Q5, respectively. Pyrazine‐derivative compounds contribute to baked, nutty, and roasted flavor profiles and are an important component of Chinese liquors. Twenty‐nine pyrazines have been identified in Moutai liquor by GC‐MS, among which 2‐methylpyrazine, 2,3‐dimethylpyrazine, 2,3,5‐trimethylpyrazine, and 2,3,5,6‐tetramethylpyrazine were quantified and the other semiquantified (Fan & Qian, 2006). In the present study, we identified several pyrazines, including 2,3,5,6‐tetramethylpyrazine, 2,3,5‐trimethylpyrazine, and 2,5‐dimethyl pyrazinel, produced by the three strains, with 2,3,5,6‐tetramethylpyrazine content reaching the highest percentages (30.737% and 33.152%) from strains Q1 and Q2, respectively, and 2,5‐dimethyl pyrazinel and 2,3,5,6‐tetramethylpyrazine reaching 26.902% and 10.638%, respectively.

**Table 3:** Volatile flavor profiles in soy sauces fermented by strains Q1, Q2, and Q5 after a 144‐hr fermentation

No.	Retention time	Volatile compounds	Content (%)		
			Q1	Q2	Q5
1	3.133	L‐Cystine	0.671	ND	ND
2	3.494	sec‐Butylamine	ND	ND	1.519
3	3.588	Aconitic anhydride (6CI)	0.501	ND	ND
4	7.703	2,3‐Butanedione	9.135	7.879	ND
5	8.054	Butane‐2,3‐dione	ND	ND	3.059
6	13.072	(+)‐(4R)‐Limonene	ND	2.056	0.851
7	13.090	Oxazole, 2,4,5‐trimethyl	2.283	ND	ND
8	14.963	3‐Hydroxy‐2‐butanone	22.976	35.463	40.965
9	15.452	Ethyl methoxyacetate	ND	ND	1.961
10	15.555	2,3‐Butanediol	0.563	0.369	4.392
11	15.757	2,5‐Dimethyl pyrazinel	4.514	7.547	26.902
12	17.292	2,3,5‐Trimethylpyrazine	7.924	9.762	8.415
13	18.516	2,3,5,6‐Tetramethylpyrazine	30.737	33.152	10.638
14	19.401	Benzaldehyde	19.063	2.236	ND
15	19.969	2‐butylcyclohexanol	0.155	ND	ND
16	20.457	1,3‐Dimethoxypropane	1.007	ND	ND
17	26.137	Phenol	ND	1.326	1.287
Subtotal			99.53	99.79	99.99

In addition to pyrazines, the strains produced high concentrations of HB, and important compound in Chinese liquors (Table 3). The metabolic activities of microorganisms during solid‐state fermentation of Chinese liquor generate a variety of precursors, including α‐acetolactate, HB, free amino acids, and ammonia, capable of converting metabolites into pyrazines via non‐enzyme‐catalyzed reactions (Rizzi, 1988). HB combined with ammonia can form 2,3,5,6‐tetramethylpyrazine at 22°C (Rizz, 1972), and HB has been detected in Chinese soy sauce liquors(Fan & Qian, 2006).

### 3.6 Effect of fermentation time and temperature on volatile flavor profiles produced by strain Q2

Table 4 shows the effect of fermentation time on Q2‐produced flavor profiles. Fourteen different volatile compounds were detected in soy sauces fermented by strain Q2, with the total volatile flavor profiles showing increasing trends during fermentation. Specifically, we observed rapid increases in pyrazine content before 48 hr, with the highest percentages reaching 50.461% after 144 hr. However, HB content initially increased and then decreased. This might be explained by the use of HB with ammonia to form 2,3,5,6‐tetramethylpyrazine (Rizzi, 1988).

**Table 4:** Effect of fermentation time on the volatile flavor profiles produced by strain Q2

No.	Retention time	Volatile compounds	Content (%)		
			48 hr	96 hr	144 hr
1	5.487	Hexamethylcyclotrisiloxane	2.672	ND	ND
2	6.188	Ethanol	ND	1.527	ND
3	7.167	2,3‐Butanedione	ND	2.713	7.879
4	7.811	Abil K 4	38.276	ND	ND
5	11.023	Decamethylcyclopentasiloxane	29.970	ND	ND
6	12.871	(+)‐(4R)‐Limonene	ND	0.225	2.056
7	14.218	DC 246	7.799	ND	ND
8	14.883	3‐Hydroxy‐2‐butanone	12.687	61.009	35.463
9	15.695	2,5‐Dimethyl pyrazine	ND	25.444	7.547
10	17.292	2,3,5‐Trimethylpyrazine	ND	6.270	9.762
11	18.516	2,3,5,6‐Tetramethylpyrazine	ND	ND	33.152
12	19.456	(R,R)‐2,3‐Butanediol			2.236
13	20.466	(R,R)‐2,3‐Butanediol			0.369
14	26.137	Phenol		2.811	1.326

## 4 CONCLUSIONS

These results confirmed that *Bacillus* strains Q1, Q2, and Q5 were able to improve the soy sauce fermentation process and promote the formation of desirable aroma components. The high content of pyrazines in soy sauces fermented by strains Q1, Q2, and Q5 demonstrated its ability to produce soy sauce. Previous studies suggested pyrazines as the most important aroma compounds for the production of soy sauce‐style liquor (Shen, 2003). In the present study, we found that 2,3,5,6‐tetramethylpyrazine content peaked at 33.152% during fermentation with strain Q2, which agreed with a previous study reporting that Maotai liquor harbors a high concentration of 2,3,5,6‐tetramethylpyrazine(Fan & Qian, 2006). In the present study, strains Q1, Q2, and Q5 promoted the formation of desirable aroma components, including HB and pyrazines, thereby enhancing the quality of soy sauce and relatively shortening the fermentation time. Our findings showed that *Bacillus* strains Q1, Q2, and Q5, and especially strain Q2, exhibited excellent fermentation ability and can be potentially used for soy sauce fermentation.

## CONFLICT OF INTEREST

The authors declare that they have no conflict of interest.

## ETHICAL APPROVAL

The study did not involve any human or animal testing.

## INFORMED CONSENT

Written informed consent was obtained from all study participants.
