# Supplementary figures and images for: Study on metabolites of Bacillus producing soy sauce‐like aroma in Jiang‐flavor Chinese spirits
Source: Food Sci Nutr. 2019 Nov 25;8(1):97–103. doi: 10.1002/fsn3.1266 (PMC6977470; doi:10.1002/fsn3.1266)

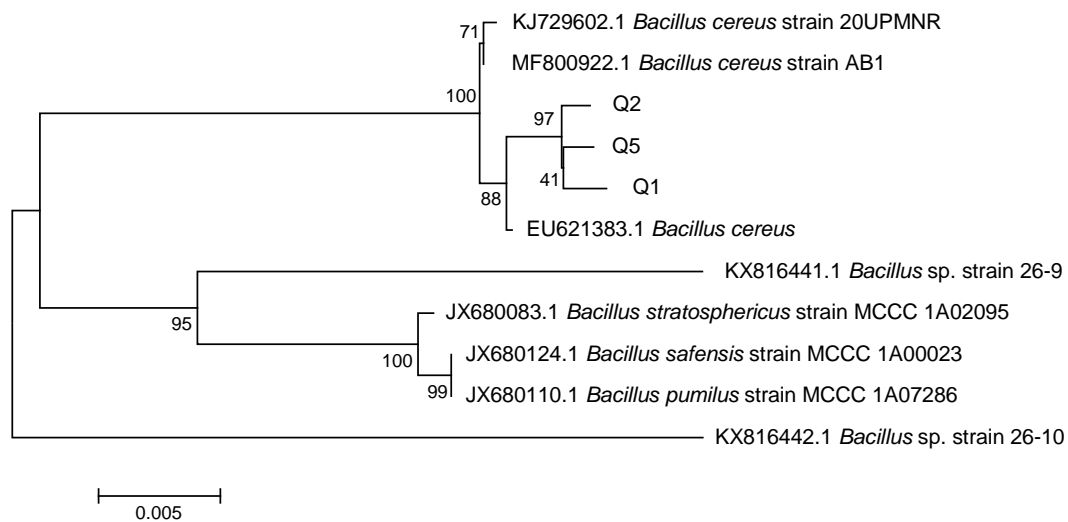

Supplement: Supplementary file 1 [file FSN3-8-97-s001.pdf]
